# Biomedical Nanosystems for In Vivo Detoxification: From Passive Delivery Systems to Functional Nanodevices and Nanorobots

**DOI:** 10.32607/actanaturae.15681

**Published:** 2023

**Authors:** T. N. Pashirova, Z. M. Shaihutdinova, V. F. Mironov, P. Masson

**Affiliations:** Arbuzov Institute of Organic and Physical Chemistry, FRC Kazan Scientific Center of RAS, Kazan, 420088 Russian Federation; Kazan (Volga Region) Federal University, Kazan, 420008 Russian Federation

**Keywords:** detoxification, nanodevices, delivery systems, enzyme

## Abstract

The problem of low efficiency of nanotherapeutic drugs challenges the creation of new alternative biomedical nanosystems known as robotic nanodevices. In addition to encapsulating properties, nanodevices can perform different biomedical functions, such as precision surgery, in vivo detection and imaging, biosensing, targeted delivery, and, more recently, detoxification of endogenous and xenobiotic compounds. Nanodevices for detoxification are aimed at removing toxic molecules from biological tissues, using a chemical- and/or enzyme-containing nanocarrier for the toxicant to diffuse inside the nanobody. This strategy is opposite to drug delivery systems that focus on encapsulating drugs and releasing them under the influence of external factors. The review describes various kinds of nanodevices intended for detoxification that differ by the type of poisoning treatment they provide, as well as the type of materials and toxicants. The final part of the review is devoted to enzyme nanosystems, an emerging area of research that provides fast and effective neutralization of toxins in vivo.

## INTRODUCTION

For a long time, human disease prevention and treatment had mostly been based on the administration of chemical or biological drugs. Since the discovery of first liposomal systems in 1964, the current nanomedicine strategy has focused on encapsulating and stabilising small molecule drugs or macromolecules in various types of nanocarriers to overcome biological barriers, increase bioavailability, reduce unwanted toxicity to healthy tissues, and target delivery [1, 2]. Despite the fact that a number of nanotherapeutic drugs have already been approved for clinical uses and/or are undergoing clinical studies [3, 4], nanomedicine still faces low efficiency in many for example, only 0.7% of cytotoxic drugs encapsulated into nanocarriers reach solid tumors [5]. Since 2008, there has been a significant increase in publications describing the production of new-generation nanotherapeutic drugs called "smart nanocarriers" that have been modified with various ligands to provide targeted delivery and sensitivity to various stimuli [6, 7].

Today, there is a demand for alternative biomedical systems such as robotic nanodevices. Unlike traditional passive nanotherapeutic drugs, these robotic nanodevices perform various biomedical functions, including precision surgery, biosensing, in vivo detection and imaging, targeted drug delivery, and, recently, detoxification [8, 9]. For a long time, nanorobotics remained a fantasy. The concept of microscopic mechanical surgeons moving through a blood vessel was first put forward in 1959 by Richard Feynman, a Nobel Prize winner in physics. Shortly after, in 1966, the concept of "surgeon" was introduced in the science fiction film Fantastic Voyage. In the film, a miniature submarine was used to remove a clot from a blood vessel. Over the past few decades, science fiction has become a reality. Nanodevices of various shapes and sizes have been developed using different types of materials, technologies, and control methods. They are often referred to as micro/nanomotors [10], micro/nano swimmers [11], micro/nano machines [12], micro/nano pumps [13], micro/nano rockets [14], etc [15].

There are many definitions for nanodevices. Nanomachines are nanoscale mechanical devices able to transform energy into precise mechanical motion [16]. Micro/nano biomedical devices often characterize structures that can be controlled and propelled within a living organism through chemical or bio-hybrid sources [17]. They are tiny nanomaterial-based integrated structures engineered in a way so that they can move autonomously and perform programmed tasks efficiently even at hard-to-reach organ/tissues/ cellular sites [18]. In summary, robotic nanodevices are next-generation tools propelled and/or guided by endogenous and exogenous stimuli for targeted and personalized therapeutic applications. However, their use for practical clinical applications is still in its infancy [19]. For the development and successful applications and clinical use of biomedical therapeutic nanodevices, the following key factors must be considered:

• biocompatibility with the patient’s body;

• ability to load/release drugs, imaging agents, etc.;

• motion control and tracking in real time using medical imaging techniques;

• controlled degradation without any toxic metabolite formation in the patient’s body

The nomenclature of micro/nanodevices is based on their design, geometry, mechanism of motion and rotation. As a rule, their self-propulsion is provided by:

a) The conversion of chemical and enzymatic [20, 21] reactions into mechanical actions [22]. Such nanodevices move in a certain direction using the energy of enzymatic or various chemical reactions [23, 24]; e.g., i) nanodevices moved by gas-bubble formation (hydrogen, oxygen, etc.); ii) self-electrophoresis-propelled nanodevices operating on the principle of redox potential difference; iii) self-diffusion nanodevices moving thanks to a concentration gradient;

b) The influence of external stimuli (magnetic, acoustic, light field); i.e., they are stimulus-sensitive nanodevices [25];

c) Biological/biohybrid nanodevices, whose movement is generated by microorganisms and cellular components such as cilia, flagella, etc [26, 27, 28, 29].

More recently, biomedical nanosystems designed for detoxification/neutralization have started to be explored. They are able to capture toxic molecules and reduce their concentration in an organism thanks to their large surface area and high affinity for active ingredients. Such systems have been designed to treat tumor and inflammatory diseases [30, 31, 32], drug overdoses [33], xenobiotic detoxification, including industrial toxicants and chemical warfare agents, etc. Typically, drug delivery systems aim at encapsulating therapeutic agents and release them in target tissues under external stimuli control. A completely opposite approach is assumed for detoxification nanodevices. These nanocarriers ensure the removal of drugs and xenobiotics from biological tissues [34]. This review provides the proof of concept and potential applications of micro/nanodevices for detoxification.

## TYPES OF NANODEVICES FOR DETOXIFICATION IN MEDICINE

Based on the general principles of therapy, detoxification is administered using:

a) antidote therapy or toxic-compound neutralization;

b) accelerated toxin elimination (hemodialysis, peritoneal dialysis, and hemosorption); and

c) symptomatic therapy; i.e., restoration of impaired functions.

**Nanosystems as nonspecific antidotes**

At present, the so-called antidotes, compounds and formulations capable of preventing or reducing the side effects of an overdose of drugs, are in demand. Nonspecific antidotes such as lipid emulsions, liposomes, and nanosponges are effectively capable of capturing drug molecules through nonspecific interactions (hydrogen bonds, hydrophobic effect, electrostatic interactions). Thus, non-specific antidotes have a broad spectrum of action for detoxification and drug overdose treatment.

Nanoemulsions. Lipid emulsion resuscitation therapy is recommended for the treatment of lipophilic drug overdoses; specifically, lipid emulsions (LE) are administered intravenously as non-specific antidotes, in the form of an "oil-in-water" type of drops [35]. LEs have been used for overdose treatment and to reduce the concentration of lipophilic antiarrhythmic, psychotropic, antimalarial drugs, local anesthetics, calcium channel blockers such as propranolol [36], cocaine [37, 38], diltiazem [39], buprenorphine, fentanyl and butorphanol [40], bupivacaine [41], ivermectin [42, 43], and ropivacaine [44, 45]. LEs rapidly decrease the threshold for seizure activity, amoxapine toxicity [46], and improve cardiac activity during heart transplantation [47]. They are also administered in cases of acute poisoning with neurotoxic organophosphorus compounds [48]. In a recent case, active toxic molecules have been removed from biological tissues using a lipophilic amine nanocarrier that is able to react with the toxin (cargo-aldehyde) inside the LEs, forming a lipophilic imine conjugate in the oil core. Successful elimination of highly toxic aliphatic aldehyde 4-hydroxynonenal from living cells is evidence in favor of the concept of living cells detoxification [34].

**Fig. 1:**
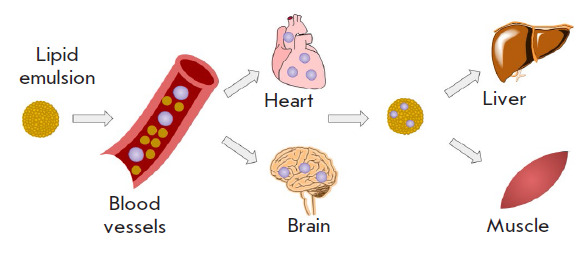
LE action mechanism in human body: the toxins are captured by lipid emulsion in high-perfusion organs, such as the heart and brain, to be transported to the liver and muscles for further redistribution. Adapted from [49]

LE action mechanism in human body is shown in Fig. 1. The emulsion captures highly lipid-soluble drugs from highly perfused organs such as the heart, brain, and kidneys and then transports them to the liver and muscles, contributing to enhanced toxins redistribution.

Recently, a dynamic multimodal LE action mechanism has been introduced. In this case, LEs capture not only toxins/drugs, but also change their pharmacokinetic profiles, exhibit a post-conditioning effect along with cardiotonic and vasoconstrictive properties, have a positive inotropic effect, reduce the release of nitric oxide, weaken mitochondrial dysfunction, phosphorylation of kinase-3β-glycogen synthase, etc. [50]. The effect of LE on the pharmacokinetic characteristics of drugs could be a guide for their clinical applications [33]. Despite the fact that LEs relieve a wide range of lipophilic drug intoxications, nevertheless, their optimal dosage, duration of administration, treatment initiation and administration have not been determined yet [51].

Nanocapsules. For the purpose of detoxification, nanocapsules (oil core/silica shell) have been synthesized [52]. The authors of [52] found that nanocapsules of smaller diameter absorbe toxins more efficiently than larger nanocapsules. The distribution of drug/toxin in nanocapsules is proportional to the area of interfacial surface and does not depend on the concentration of oil phase. In addition, the drug distribution decreases when the thickness of shell increases, since there is a decrease of drug penetration into the nanocapsules with a thicker shell [52]. For the treatment of alcohol intoxication, nanocapsules imitating hepatocyte detoxifying functions were developed to deliver enzymes (alcohol oxidase, catalase, and aldehyde dehydrogenase) to the liver. Alcohol oxidase and catalase contributed to the rapid removal of alcohol. The resulting acetaldehyde was effectively oxidized by aldehyde dehydrogenase. Administration of the developed antidote to mice suffering alcoholic intoxication provided a significant decrease alcohol concentration in the bloodstream without acetaldehyde accumulation [53].

**Fig. 2:**
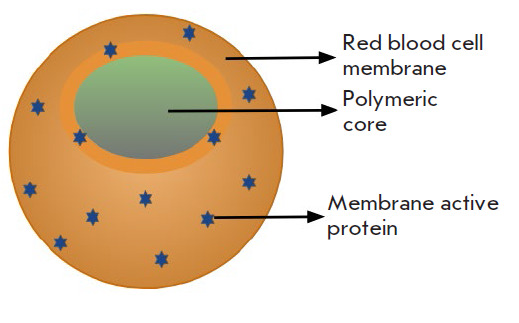
Nanosponge structure that consists of a polymer-covered core with an erythrocyte membrane shell. Redrawn and modified from [55]

Nanosponges. Nanosponges are a naturally degradable 3D scaffold formed in solution by crosslinkers. [54]. For the first time, nanosponges covered by a natural cell membrane and functioning through biomimicry were proposed by Zhang L. in [55]. "The nanosponge acts as a toxin bait in vivo and is a new way to remove toxins from the bloodstream", Zhang L said. "Instead of building specific products to treat individual toxins, we are developing a platform that can neutralize the toxins produced by a wide range of pathogens". The nanosponges developed by Zhang L and colleagues consist of poly(lactic-co-glycolic acid) polymer core (PLGA) and an outer shell of red blood cell membrane that attract toxins like a bait (Fig. 2).

In tests on mice, prophylactic administration of nanosponges reduced mortality down to 11%, compared to 100% mortality without treatment. With nanosponges, mortality in mice dropped to 56% after toxin injection. Suggestively, the nanosponges containing the isolated toxin accumulated in the liver, where, in the absence of any damage, the toxin was safely metabolized and eliminated from the body [55, 56].

Nanosponges were effectively used for detoxification of bacterial toxins [57, 58]. They were able to bind and neutralize low-molecular-weight compounds [59], autoimmune antibodies [60], inflammatory cytokines [61], bacteria and viruses [62, 63], and neurotoxins (tetrodotoxin, botulinum toxin, and saxitoxin) [64]. Nanosponges for the neutralization of neurotoxins consist of a polymer core covered by a membrane of neurons; namely, neuro-2a cells. The use of this mouse neural crest-derived cell line increased the mice’s survival rate in the absence of acute toxicity [64]. Two-modal detoxification with nanosponges that possess an oil core and are coated with an erythrocyte membrane (Oil-NS) was more effective [65]. This Oil- NS construction combines the specific binding capacity of biological receptors on the cell membrane with the non-specific absorption of oil core. Together, they increase the overall detoxification capacity.

A nanosponge-gel hybrid system also neutralizes toxins. Its use for both therapeutic and prophylactic purposes has led to a significant improvement in the treatment of toxin-related skin damage [66]. A subject for further study is a biomimetic detoxification strategy based on the creation of nanoparticles coated with a platelet membrane. Such systems can be promising as additional therapy for patients with a methicillin-resistant Staphylococcus aureus infection [67].

Erythroliposomes. Erythroliposomes (RM-PL) are a biomimetic platform consisting of artificial lipid membranes and natural erythrocyte membranes. Such systems have been successfully used to neutralize various hemolytic pore-forming toxins [68]. The toxins absorbed by RM-PL are transferred to the liver and spleen, where they undergo endocytosis and are digested by macrophages. In mice, administration of RM-PL eliminates initial toxicity in target organs, allowing the animals to survive.

**Fig. 3:**
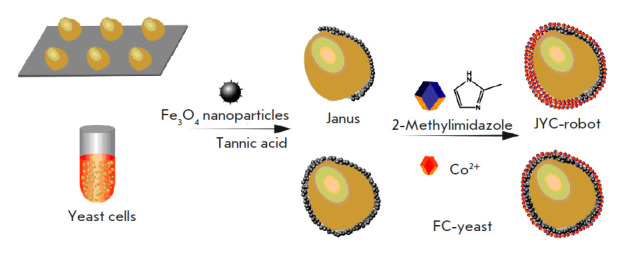
Designing biomimetic hybrid systems for the neutralization of mycotoxins. Adapted from [73]

Biomimetic hybrid systems. Janus micromotors are magnesium and gold particles coated with a red blood cell membrane (RBC-Mg) that acts as a bait and absorbs and neutralizes biological toxins in water and biological media. RBC-Mg nanomotors have been used to rapidly detoxify α-toxin and methylparaoxon, models of membrane-damaging toxins and chemical warfare agents, respectively [69, 70]. Hybrid biomembrane nanorobots with an acoustic drive and a membrane consisting of two types of cells (erythrocytes and platelets) effectively bind to both toxins and pathogens in the blood. To eliminate simultaneously pathogenic bacteria and toxins the proteins located in the hybrid membrane are used. They bind to pathogens and neutralize pore-forming toxins [71, 72]. There are examples [73] of microrobots with Fe_3_O_4_ nanoparticles covering yeast cells and creating a zeolite imidazolate framework-67 (ZIF-67) to neutralize mycotoxins (Fig. 3).

**Nanodialysis systems for improved detoxification**

**Fig. 4:**
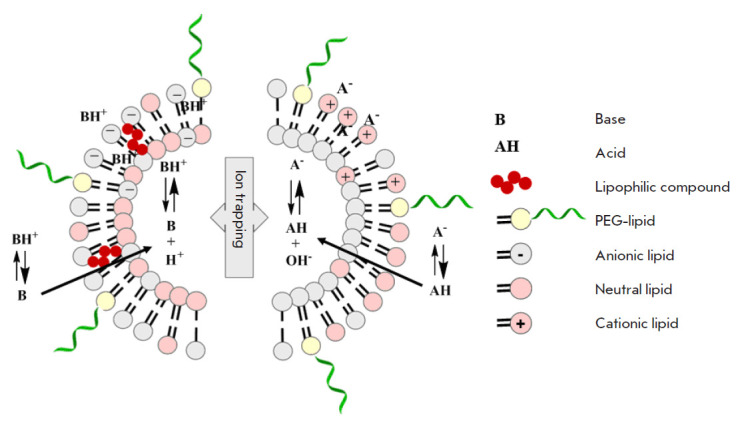
pH-gradient liposomes with a pH gradient between the internal and external environments: acidic/basic pH inside, neutral pH outside. Adapted from [82]

Liposomes. Designing liposomal dialysates is an emerging area of research. Liposomes without drugs, "empty liposomes", were used as scavengers for exogenous and endogenous toxic molecules. Some of these studies have reached clinical trials. It is quite possible that liposomes will be medically used as nanoantidotes in the next decade [74]. The introduction of "empty" liposomes contributes to reservoir formation for toxin binding. Liposomes bind toxins through electrostatic interactions and a hydrophobic effect in the membrane or through ion trapping into the hydrophilic core. Non-ionized molecules penetrate the liposome membrane. They are captured by a hydrophilic, pH-controlled core of liposomes. For example, a weakly basic drug molecule, upon entering a hydrophilic core with an acidic pH value can be ionized and lose its ability to diffuse through the lipid bilayer of liposome membrane (Fig. 4).

For the first time, a hemodialysis method including liposomes and antioxidants has been presented as a unique strategy for removing toxins. Its application in vitro resulted in a further noticeable decrease in the amount of oxidation products and removal of platelets and bilirubin when compared to conventional hemodialysis [75]. In vivo experiments in rats suffering from uremia confirmed that the addition of liposomes to the dialysate as an adjunct to conventional hemodialysis facilitated the removal of protein-bound uremic solutes. The developed nanosystem has unique advantages in comparison with albumin and other alternatives that use sorbents [76]. Modified by linoleic acid [77] and decorated with polyethyleneimine, liposomes demonstrated significantly higher binding rates and rapid clearance of protein-bound uremic toxins [78]. Preclinical evaluation of transmembrane liposomes with a pH gradient for the ammonia concentration confirmed the ability of liposome-supported peritoneal dialysis (LSPD) to reduce plasma ammonia levels in pigs with artificially induced hyperammonemia [79]. LSPD, in particular its peritoneal dialysate, enriched with pH-gradient liposomes, i.e., with a pH gradient between the internal and external environments of liposomes (acid inside, neutral outside), alleviated the symptoms of poisoning in animal models in [80, 81]. An apparent increase in the concentrations of haloperidol, verapamil, and amitriptyline in the dialysate using LSPD was observed in rats compared to a peritoneal dialysate without augmentation [80, 81]. LSPD was used to remove toxins/highly plasma protein bound drugs. Amitriptyline was chosen as a drug that highly binds to plasma proteins. It was shown that LSPD increases amitriptyline extraction in vivo in [82].

**Table 1:** Types of detoxification nanodevices, materials and enzyme/drug library

Nanodevices	Material	Neutralization	In vivo model	Ref.
LE	Lipoamine	Cargo-aldehyde	-	[34]
	Intralipid	Propranolol	White rabbits	[36]
	Intralipid	Cocaine	Clinical trial	[37]
	Intralipid	Cocaine	Dog	[38]
	Intralipid	Diltiazem	Clinical trial	[39]
	Intralipid	Buprenorphine, fentanyl, butorphanol	-	[40]
	Intralipid	Bupivacaine	Pigs	[41]
	Intralipid	Ivermectin	Pogona vitticeps	[42]
	Intralipid	Ropivacaine	Pigs	[44]
	Intralipid	Sevoflurane, isoflurane	Rats	[45]
	Intralipid	Amoxapine	Clinical trial	[46]
	Intralipid	Organophosphates	Clinical trial	[48]
Nanocapsules	Tetramethoxysiloxane, octadecyltrimethoxysilane, ethyl-butyrate, lecithin, Tween-80	Quinoline	-	[52]
	Acrylamide, APm, N,N’–methylenebisacrylamide, enzymes (Alcohol oxidase, Catalase, Aldehyde dehydrogenase)	Ethanol	C57BL/6 mice	[53]
Nanosponges	RBC membrane, PLGA	Bacterial toxins (melittin, α-hemolysin, listeriolysin O, streptolysin O)	-	[57]
	RBC membrane, PLGA	Bacterial toxins	CD-1 mice	[58]
	RBC membrane, PLGA	Bichlorvos	CD-1 mice	[59]
	RBC membrane, PLGA	Autoantibodies	CD-1 mice	[60]
	Peripheral blood neutrophils membrane, PGLA	Proinflammatory cytokines	ICR mice	[61]
	Bacterial membrane, PLGA	Bacteria	C57BL/6 mice	[62]
	Lung epithelial cells membrane/macrophage membrane, PLGA	SARS-CoV-2	C57BL/6NHsd mice	[63]
	Neuro-2a cells membrane, PLGA	Tetrodotoxin	ICR mice	[64]
	RBC membrane, olive oil	Organophosphates (paraoxon, diisopropyl fluorophosphate, dichlorvos)	ICR mice	[65]
	RBC membrane, PLGA, Pluronic F127	Pore-forming toxins	ICR mice	[66]
	Platelet membrane, PLGA	S. aureus	CD-1 mice	[67]
Erythroliposomes	RBC membrane, cholesterol, phosphatidylcholine, mPEG-DSPE EPC,	Pore-forming toxins	ICR mice	[68]
Janus micromotors	RBC membrane, Mg, Au, chitosan	α-toxin	-	[69]
	RBC membrane, Au, citric acid	Melittin	-	[70]
Hybrid biomembrane nanorobots	RBC membrane, Au	Pore-forming toxins	-	[71]
	RBC and platelet membrane, Au	Pore-forming toxins	-	[72]
Janus microrobots	Yeast cell membrane, Fe_3_O_4_, 2-methylimidazole	Mycotoxins	-	[73]
Liposomes	Lecithin, cholesterol, deoxysodium cholate	Protein-bound uremic toxins	Sprague Dawley rats	[76]
	Lecithin, cholesterol, linoleic acid, Tween-80	Protein-bound uremic toxins	-	[77]
	Lecithin, cholesterol, linoleic acid, polyethylenimine, Tween-80	Protein bound uremic toxins	-	[78]
LSPD	DPPC, cholesterol, mPEG-DSPE, citric acid	Ammonia	Göttingen minipig	[79]
	DPPC, cholesterol, DSPE-mPEG	Ammonia	Sprague Dawley rats	[80]
	DPPC, cholesterol, DSPE-mPEG	Amitriptyline	Sprague Dawley rats	[81]
	DOPG, cholesterol	Amitriptyline	Sprague Dawley rats	[82]
	Phosphatidylcholine, cholesterol, DSPE-mPEG, iron citrate	Phosphate ions	-	[83]
	DOPE-NHS, β-octylglucoside, enzymes (Alcohol oxidase, Catalase)	Ethanol	Sprague Dawley rats	[84]

Polyethylene glycol-modified liposomes encapsulated with phosphate-binding iron (III) citrate trap the circulating phosphate ions into the inner liposomal core. These traps reduce the concentration of free phosphate ions in solution and in serum [83] (Table 1).

## ENZYME NANODEVICES FOR DETOXIFICATION

A detailed description of nanoparticles with encapsulated enzymes is present in our recently published review [85], where nanoparticle types, materials, results of clinical studies, etc., were considered. Therefore, in this part of this review, we want to focus on such systems as enzymatic nanodevices for neutralizing toxins. Enzyme encapsulation in nanocarriers opens up possibilities for the creation of nanoreactors, nanodevices that contain the molecules needed to support anomalous diffusion and abide by kinetic laws. Such systems are capable of performing single or cascade reactions either for biosynthesis or for degradation of toxic substrates [86]. Nanobiotechnology of enzyme nanoreactors is a new, rapidly developing area of research. For example, recently, alcohol oxidase and catalase enzymes-containing liposomes supporting peritoneal dialysis have been investigated. In this nanoreactor, H_2_O_2_ addition accelerates ethanol removal for H_2_O_2_, to be rapidly decomposed into O_2_ by the catalase, while enzymatic liposomes enhance ethanol metabolism. In a model of rodent intoxication with ethanol, enzyme liposomes enhanced ethanol metabolism, as was evidenced by increased production of acetaldehyde, the main metabolite of ethanol [84].

In our laboratory, we focus on the design and development of injectable therapeutic enzyme nanoreactors for the neutralization of toxicants like organophosphorous (OP) pesticides [87]. Enzymes capable of detoxifying OPs can be used as bioscavengers. They act either as stoichiometric, pseudocatalytic or catalytic traps for OP molecules [88, 89]. These enzymes, phosphotriesterases and cholinesterases, are the active components of these therapeutic nanodevices. Encapsulation of bioscavengers in such vehicles is first intended to overcome the fast clearance and immune response after injection of soluble heterologous therapeutic enzymes. The aim of enzyme encapsulation into nanoreactors is also to provide a high concentration of reactive enzyme in stable nanocontainers. Determining the concentration of the encapsulated enzyme inside nanocarriers is an important step in designing an efficient in vivo nanoreactor. In presence of an injectable nanoreactor, the toxicant present in the bloodstream diffuses across the nanoreactor’s membrane, and the enzyme-mediated detoxification reaction takes place inside the sealed compartment [90]. Enzyme concentration (E) inside a nanobody can be either low or much higher than that of the toxicant (T). The reaction inside the nanoreactor occurs either under first ((E) < < (T)) or second-order conditions ((E) ≈ (T)) with respect to toxicant concentration (T). However, partial enzyme encapsulation may occur as well and, in this case, an enzymatic "corona" forms on the outer surface of the nanoreactors, which can complicate the process and lead to the undesirable, rapid clearance and possible adverse immune responses to heterologous enzymes. Thus, the permeability of the nanoreactor membrane for substrates and reaction products, possible osmotic effects, the effects of viscosity and crowding, and the formation of an enzymatic "corona" are important technological problems that have not yet been fully resolved. [90].

## CONCLUSION

The number of publications devoted to the development of alternative, efficient, and multifunctional biomedical systems such as robotic nanodevices for detoxification is on the rise. This review shows that the proposed concept of nanodetoxification requires an interdisciplinary approach and the borrowing of knowledge from many different fields, such as nanosystem design, biochemistry, biotechnology, micro-and optoelectronics, etc. One of the possible directions in acute poisoning treatment is the development of "empty" nanomedical preparations based on materials and compounds that have been approved for clinical use. In addition, designing nanodevices opens up new opportunities in the treatment of bacterial and viral infections.

However, there is still a long way before highly sensitive, easily controlled, and safe nanodevices are created and such problems as moving in narrow and hard-to-reach places (e.g., capillary blood vessels), performing complex functions, being flexible and cost-effective are resolved.

## Abbreviations

LE: lipid emulsions
RM-PL: erythroliposome
LSPD: peritoneal dialysis with liposomes
OP: organophosphorus compounds
E: enzyme
T: toxicant
